# Correction: Effects of dysmenorrhea on work productivity and quality of life in Japanese women: A large-scale web-based cross-sectional study

**DOI:** 10.1371/journal.pone.0340615

**Published:** 2026-01-06

**Authors:** Maika Nariai, Osamu Wada-Hiraike, Eri Maeda, Masayo Matsuzaki, Mayuyo Mori-Uchino, Maho Furukawa, Yuki Enomoto, Hiromi Ga, Risa Takai, Miyuki Harada, Yutaka Osuga, Yasushi Hirota

There are errors in the Funding statement. The correct Funding statement is as follows: This study was supported by the Ministry of Health, Labour and Welfare (24FB1001: OWH, No number and 23FB1001: YO).

## References

[1] Nariai M, Wada-Hiraike O, Maeda E, Matsuzaki M, Mori-Uchino M, Furukawa M, et al. Effects of dysmenorrhea on work productivity and quality of life in Japanese women: A large-scale web-based cross-sectional study. PLoS One. 2025;20(11):e0329154. doi: 10.1371/journal.pone.0329154 41284693 PMC12643315

