# Supplementary figures and images for: Effects of dysmenorrhea on work productivity and quality of life in Japanese women: A large-scale web-based cross-sectional study
Source: PLoS One. 2025 Nov 24;20(11):e0329154. doi: 10.1371/journal.pone.0329154 (PMC12643315; doi:10.1371/journal.pone.0329154)

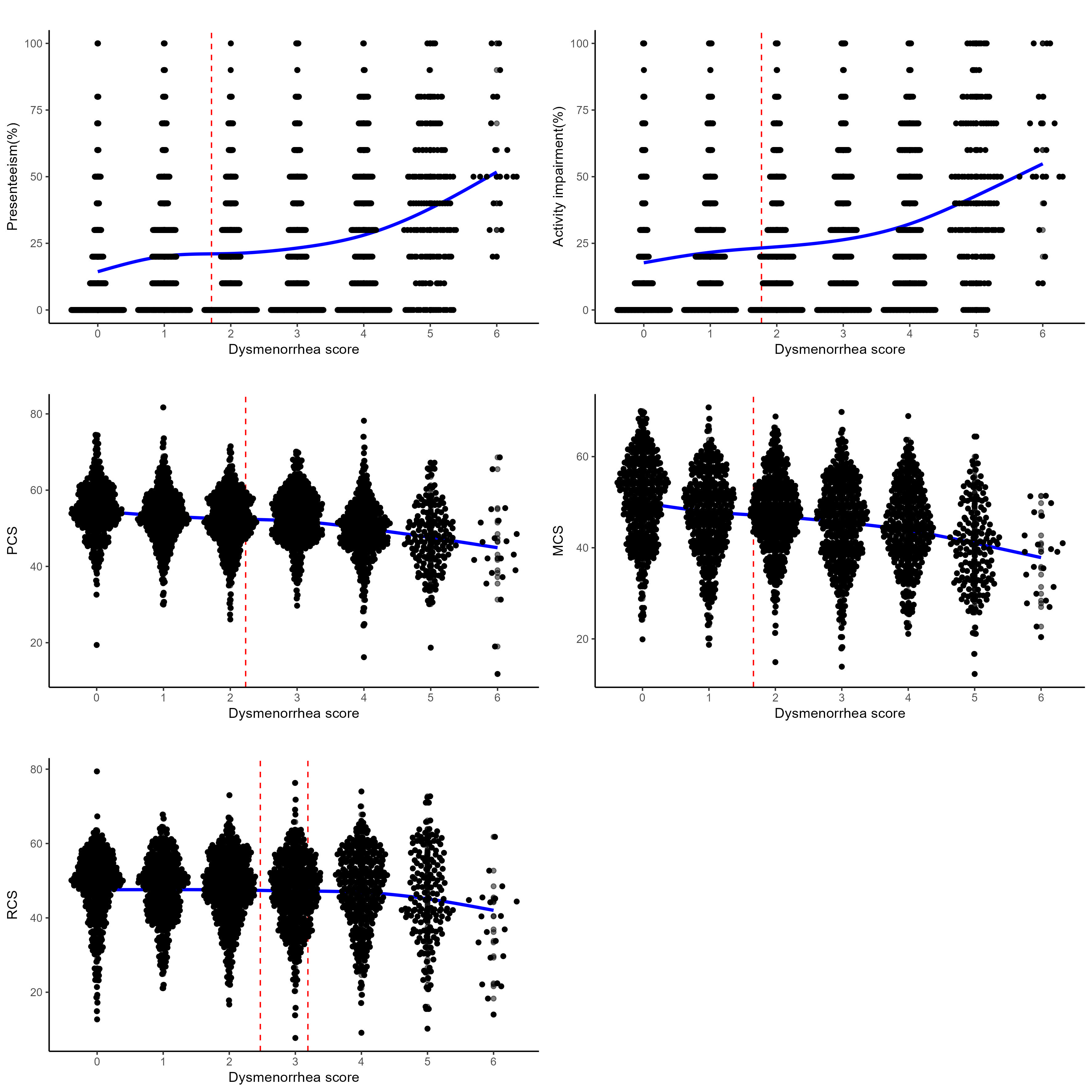

Supplement: S1 Fig — The blue curve represents a quartic polynomial fit, and the red dashed lines indicate the positions of the inflection points. For absenteeism, no clear inflection point was found and thus omitted. (JPG) [file pone.0329154.s001.jpg]
